# Correction: MMP-3 cleavage of Lamin A induces pro-migratory nuclear deformity, nucleophagy, and their autophagic secretion with extracellular vesicles in metastatic cancer

**DOI:** 10.1186/s12964-026-02815-1

**Published:** 2026-03-18

**Authors:** Takanori Eguchi, Eman A. Taha, Keisuke Nakano, Vikas Tiwari, Katsuki Takebe, Tomohiro Inoue, Lizi Xing, Chiharu Sogawa, Kuniaki Okamoto, Stuart K. Calderwood

**Affiliations:** 1 Department of Dental Pharmacology, Faculty of Medicine, Dentistry and Pharmaceutical Sciences, Okayama University, Okayama, 700-8525 Japan; 2 Department of Biochemistry, Faculty of Science, Ain Shams University, Cairo, 11566 Egypt; 3 Department of Oral Pathology and Medicine, Faculty of Medicine, Dentistry and Pharmaceutical Sciences, Okayama University, Okayama, 700-8525 Japan; 4 Council of Scientific & Industrial Research-Indian Institute of Toxicological Research, Lucknow, 226001 India; 5 Research practicum program, Okayama University Dental School, Okayama, 700- 8525 Japan; 6 Department of Clinical Pharmacology, Harbin Medical University Cancer Hospital, Harbin, 150040 China; 7 Department of Food and Health Sciences, Faculty of Environmental Studies, Hiroshima Institute of Technology, Hiroshima, 731-5193 Japan; 8 Division of Molecular and Cellular Biology, Department of Radiation Oncology, Beth Israel Deaconess Medical Center, Harvard Medical School, Boston, MA 02115 USA

**Correction: Eguchi et al. Cell Communication and Signaling 24, 146 (2026)**

**https://doi.org/10.1186/s12964-025-02549-6**

Following publication of the original article [1], the authors requested to replace Fig. 6 which was mistakenly processed (similar to Fig. 5) by the production team.

The original article [1] has been corrected.
